# Mouse whole embryo culture: Evaluating the requirement for rat serum as culture medium

**DOI:** 10.1002/bdr2.1538

**Published:** 2019-06-24

**Authors:** Lucy H. Culshaw, Dawn Savery, Nicholas D. E. Greene, Andrew J. Copp

**Affiliations:** ^1^ Newlife Birth Defects Research Centre, UCL GOS Institute of Child Health University College London London United Kingdom

**Keywords:** birth defects, development, embryo culture, neural tube, organogenesis, postimplantation, teratogen, yolk sac

## Abstract

**Background:**

Whole embryo culture is a valuable research method in mammalian developmental biology and birth defects research, enabling longitudinal studies of explanted organogenesis‐stage rodent embryos. Rat serum is the primary culture medium, and can sustain growth and development over limited periods as in utero. However, the cost, labor, and time to produce culture serum are factors limiting the uptake of the methodology. The goal of replacing or at least reducing rat serum usage in culture would be in accordance with the principles of “replacement, reduction, and refinement” of animals in research (the 3Rs).

**Methods:**

We performed cultures of mouse embryos for 24 hr from embryonic day 8.5 in serum‐free media or in rat serum diluted with defined media, compared with 100% rat serum. Developmental parameters scored after culture included yolk sac circulation, dorsal axial length, somite number, protein content, and completion of cranial neural tube closure.

**Results:**

A literature review revealed use of both serum‐free and diluted rat serum‐based media in whole embryo culture studies, but with almost no formal comparisons of culture success against 100% rat serum. Two serum‐free media were tested, but neither could sustain development as in 100% rat serum. Dilution of rat serum 1:1 with Glasgow Minimum Essential Medium plus defined supplements supported growth and development as well as whole rat serum, whereas other diluent media yielded substandard outcomes.

**Conclusion:**

Rat serum usage cannot be avoided, to achieve high quality mouse embryo cultures, but rat usage can be reduced using medium containing diluted serum.

## 1 INTRODUCTION

Rodent whole embryo culture (WEC) allows direct observation and manipulation of developing mammalian embryos, outside the confines of the maternal uterus. In the early 1960s, an *ex utero* technique was described for growing postimplantation rat embryos through the stages of organogenesis (New, 1966). This work initially involved establishment of culture conditions for various 24–48 hr periods between embryonic days (E) 7.5 and 13.5, and culminated in viable cultures continuing for as long as 5 days (Buckley, Steele, & New, 1978). Assessment of somite number, degree of axial rotation, developmental progression, and protein content showed that development and growth of embryos can be closely similar in culture and in vivo, both in rats and mice (New, 1978; Sadler, 1979).

**Figure 1:**
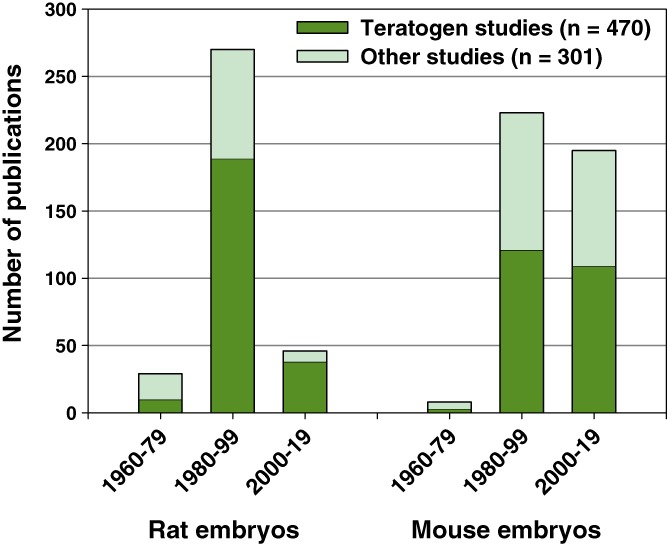
Analysis of whole embryo culture publications using rat and mouse embryos. Number of embryo culture publications in 20‐year periods since the methodology began in the early 1960s. Rat embryo studies began earlier than mouse studies, peaked during 1980–1999, and have declined in numbers more recently. Mouse studies have continued in similar numbers to the present. Teratogen‐related research comprises a greater proportion of embryo culture studies using rats than mice (rat: 68.7% of total; mouse: 54.7%; chi‐square test: *p* < .001). Indeed, the most recent rat embryo culture studies are largely (38/46) teratogen‐based

We reviewed the literature to discern trends in WEC, over the 60 year history of the method. Rat embryos were mainly cultured in the early years, but the last two decades saw a sharp decline in rat studies (Figure 1). In contrast, WEC studies with mouse embryos have continued at similar levels over the last 40 years, with around 10 new publications each year. Rat and mouse WEC studies also differ in focus: Rats have been used mainly in teratogen‐related research (i.e., to investigate exogenous influences on the embryo), whereas this is a significantly smaller component of mouse WEC studies (Figure 1). In contrast, mouse WEC is increasingly focused on developmental biology, with application of methods including electroporation of gene expression constructs (Calegari, Marzesco, Kittler, Buchholz, & Huttner, 2004; Osumi & Inoue, 2001), surgical manipulation (Angelo & Tremblay, 2013), biomechanical assessment (Hughes, Greene, Copp, & Galea, 2017), and live imaging (Massarwa & Niswander, 2013). Such studies benefit from the diverse genetic tools available in mice (Pryor, Massa, Savery, Greene, & Copp, 2012). In view of this analysis, the present study focuses on mouse embryo WEC methodology.

A feature of the WEC method is the use of specially prepared rat serum (RS) as culture medium. In particular, the blood must be centrifuged before clotting begins, otherwise developmental abnormalities result (Steele & New, 1974). These exacting preparation requirements led most laboratories to prepare their own RS in‐house, an expensive and time‐consuming activity. Many studies have tested other sera and media with the aim of finding a simple, cheap alternative to RS. Early work evaluated Waymouth's medium, Dulbecco modified Eagle's medium (DMEM), Tyrode's solution, new‐born calf serum and fetal calf serum, but all failed to support normal growth (Sadler & New, 1981). Sera from humans, monkeys, cows and dogs were also tested, but none proved as effective as RS (Chambers, Klein, Nosel, Khairallah, & Romanow, 1995; Chatot, Klein, Piatek, & Pierro, 1980; Coelho, Weber, Klein, Daniels, & Hoagland, 1989; Flynn, Friedman, Black, & Klein, 1987). Hence, the use of RS as culture medium remains standard practice in WEC (Takahashi, Makino, Kikkawa, & Osumi, 2014).

We asked whether alternatives to 100% RS have been rigorously evaluated in mouse WEC. Thirty eight publications were identified in which medium usage departed from the 100% RS standard (Table S1). Of these, 13 used sera other than RS, 22 used RS diluted with various defined media, and 4 reported use of serum‐free, completely defined media. However, only six of the 36 studies included formal comparisons of culture success against parallel cultures in 100% RS (studies 18, 26, 27, 33, 35, and 38; Table S1). Of these, four studies evaluated WEC success in heterologous sera compared with RS (Hunter III, Balkan, & Sadler, 1988; Sadler, 1979; Tam & Snow, 1980; Van Maele‐Fabry et al., 1991), while three studies compared WEC outcomes in diluted RS, compared with 100% RS. Two of these latter studies cultured embryos only up to gastrulation‐stage (Miura & Mishina, 2003; Tam & Snow, 1980). The third study compared culture success at early organogenesis stages (E8.5–9.5)(Clarkson, Doering, & Runner, 1969), but commercially prepared serum (without immediate centrifugation) was used with the conclusion that RS diluted to 10–25% supported development better than 100% RS. There have been no studies comparing completely defined (serum‐free) media with 100% RS.

In view of the importance of culture medium for WEC, and the continuing need to reduce rat usage in the interests of the 3Rs (http://www.nc3rs.org.uk/the-3rs), we undertook a study of E8.5–9.5 mouse WEC, to evaluate two serum‐free media, and dilutions of in‐house‐produced RS using various defined media. The E8.5–9.5 period of organogenesis is known to be critical for the origin of developmental anomalies that lead to clinically important birth defects; hence, embryo culture is of particular importance in enabling experimental studies at this stage. We find that serum‐free culture does not yet yield acceptable WEC results, whereas 50% dilution of RS with one specific defined medium is compatible with high quality WEC outcomes.

## 2 MATERIALS AND METHODS

### 2.1 Mouse strains, embryo collection, and RS preparation

All animal studies were performed as specified and licensed under the regulations of the UK Animals (Scientific Procedures) Act 1986, Amendment Regulations (SI 2012/3039). Animal work conformed to the UK Medical Research Council's *Responsibility in the Use of Animals for Medical Research* (1993). Random‐bred CD1 mice were mated overnight and checked for copulation plugs the next morning. Noon on the day of plug detection was designated embryonic day (E) 0.5, equivalent to 0.5 days post coitum. At E8.5, pregnant females were euthanized by cervical dislocation and embryos were dissected for culture as described (Copp et al., 2000). RS for WEC was prepared and stored as described (Cockroft, 1990; Takahashi et al., 2014). Serum aliquots were thawed at 37 °C and filtered through a 0.45 μm filter (Merck) immediately before use.

### 2.2 Serum‐free culture media

KnockOut DMEM (Gibco 10829‐018) comprised: 10% KnockOut serum replacement (KSR) (Gibco 10828010), 1X N‐2 supplement (100X stock, Gibco, 17502‐048), 2% bovine serum albumin (BSA; Sigma A9418‐50G) and 25 U/mL of penicillin‐streptomycin (Gibco, 15140‐122). BSA was first added to the KnockOut DMEM, followed by filtration through a 0.45 μm pore size filter. The final solution was mixed thoroughly and filtered once more through a 0.22 μm pore size filter.

N2B27 serum‐free culture medium comprised: 25% low glucose DMEM without phenol red (Invitrogen, 11880‐028), 25% Ham's F‐12 nutrient mix with GlutaMAX (Invitrogen, 31765), 50% Neurobasal‐A medium without Phenol Red (Gibco, 12349‐015), 2X N2 supplement (100X stock, Gibco, 17502‐048), 2X B27 supplement (50X stock, Gibco, 17504‐044), 25 U/mL of penicillin‐streptomycin (Gibco, 15140‐122), and 0.1 mM β‐mercaptoethanol (Sigma, M3148).

Components of both media were stored according to manufacturer's instructions. Medium aliquots were stored at −20° to avoid repeated thawing and refreezing.

### 2.3 Defined media for dilution of RS

DMEM comprised: DMEM with phenol red, +4.5 glucose, +L‐glutamine, +HEPES, –sodium pyruvate (Thermofisher, 42430‐025). Glasgow Minimum Essential Medium (GMEM) + defined supplements (DS) comprised: GMEM (Sigma, G5154), 1% nonessential amino acids (Life Technologies, 11140050), 2 mM L‐glutamine (HyClone™ SFM4CHO‐Utility media [liquid] with L‐glutamine) (Fisher Scientific, SH30549.01), 1 mM sodium pyruvate solution (Fisher Scientific, SH30239.01), 25 U/mL of penicillin‐streptomycin (Gibco, 15140‐122). Glucose addition experiments used: glucose (D‐[+]‐glucose SigmaUltra ≥99.5% GC) (Sigma, G7528). All serum‐free media containing multiple components were stored at 4 °C and used within 2 days. All media were gassed with 5% O_2_, 5% CO_2_, 90% N_2_, and warmed to 37 °C before embryos were added.

### 2.4 Embryo dissection and culture

Embryos were maintained in warm DMEM + 10% fetal bovine serums (FBS) throughout the dissection process. Once ready for culture, the embryos in a particular batch were ranked according to size (largest to smallest) and distributed alternately into the treatment groups. This ensured that groups for comparison began culture with as similar a mean embryonic stage as possible. Care was taken to transfer as little DMEM + 10% FBS as possible into the culture tubes. The sealed roller‐bottle technique (New, Coppola, & Terry, 1973) was used to culture E8.5 mouse embryos for 24 hr at 38 °C in a gas atmosphere of 5% O_2_, 5% CO_2_, and 90% N_2_. A volume of 0.5–1.0 mL RS, serum‐free medium or diluted RS was used per embryo in all experiments, except for the study in which medium volume was specifically reduced to 0.3 mL per embryo. Culture medium was not changed during the 24 hr culture.

### 2.5 Postculture embryo scoring

**Figure 2:**
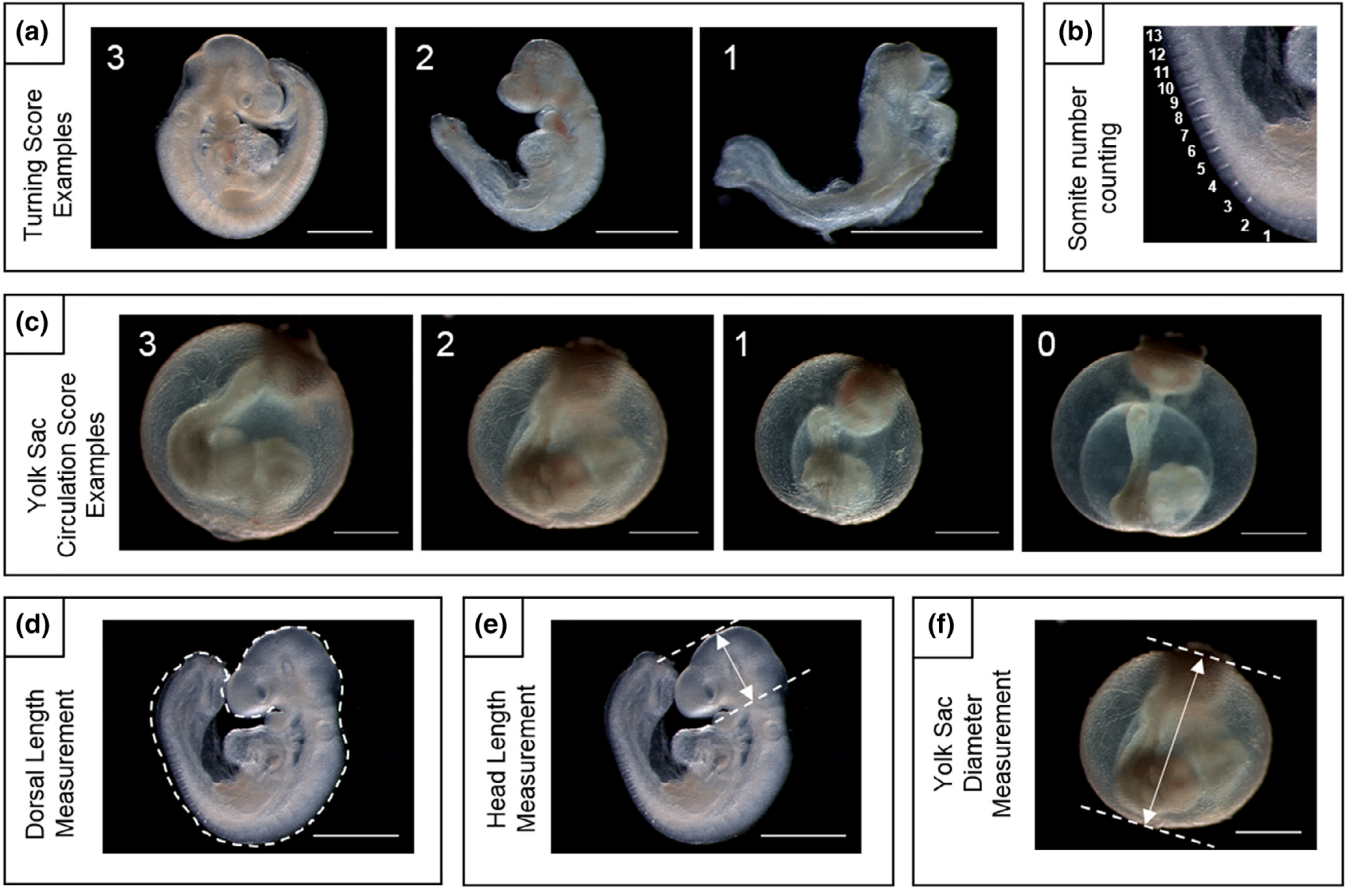
Developmental scoring and measurement techniques used in the study. (a) “Turning” (i.e., axial rotation) scores: 3—embryo with full turning, exhibiting an entirely convex dorsal surface; 2—embryo with incomplete turning: The caudal part of the body axis remains dorsally concave and is not positioned beside the head; 1—embryo with no turning, where the dorsal surface is entirely concave. (b) Somite number counting performed in a rostral to caudal direction along the dorsal length of the embryo. (c) Yolk sac circulation scores: 3—full yolk sac plexus of vessels with rapid heartbeat and pulsatile blood flow; 2—developed yolk sac vasculature and steady heartbeat but slow or intermittent blood movement; 1—blood islands and/or minimal blood movement; 0—no visible blood islands and no blood movement, with slow or infrequent heartbeat. (d) Dorsal length, as measured along the entire dorsal aspect of the embryo, from the ventral surface of the forebrain where it abuts the first branchial arch, to the caudal extremity of the body axis. Line length was measured by ImageJ. (e) Head length, measured between parallel lines tangential to the crown of the head, and to the ventral forebrain surface. (f) Yolk sac diameter, measured from the base of the ectoplacental cone to the furthest perimeter. Scale bars: 1 mm

To ensure blinding of phenotypic scoring, as specified by the NC3Rs ARRIVE guidelines (https://www.nc3rs.org.uk/arrive-guidelines), culture bottles were labeled with a code by a second party and analysis was conducted blind to treatment group (Figure 2).

After 24 hr, embryos were removed from the culture medium and transferred to warm DMEM + 10% FBS. Without removing the membranes, yolk sac circulation was scored immediately, based on the Brown and Fabro scoring system (Brown & Fabro, 1981): 0 = no visible blood islands and no obvious blood movement, with slow or infrequent heartbeat; 1 = evidence of blood islands but slow/very little blood movement; 2 = evidence of developed yolk sac vasculature and steady frequent heartbeat; 3 = full yolk sac plexus of vessels with rapid blood movement. Axial rotation was scored according to the system: 1 = ventrally convex (unturned); 2 = mid‐axial rotation in progress but incomplete (turning); 3 = dorsally convex (fully turned). Yolk sac diameter was measured from the base of the ectoplacental cone to the furthest yolk sac perimeter using a micrometer eyepiece on a stereomicroscope.

The yolk sac and amnion were then removed and head‐length was measured from the superior to inferior aspect of the head using a micrometer eyepiece. Each embryo was photographed, and dorsal length was subsequently measured using ImageJ: A freehand line was drawn and measured from the inferior aspect of the head, over the crown and continuing along the entire dorsal aspect of the embryo to the caudal tip. This dorsal length measurement was designed to remove discrepancies inherent in the traditional crown‐rump length measurement, which is not appropriate for unturned or partially turned embryos. Somite number was determined by counting in a rostral to caudal direction. Any developmental abnormalities, such as open cranial neural tube, were recorded for each embryo.

### 2.6 Protein content analysis

After phenotypic scoring, embryos were rinsed in PBS, excess liquid was removed and embryos were snap‐frozen on dry ice. RIPA buffer (100 μL per tube: 5 mM Tris‐Cl pH 7.4, 150 mM NaCl, 1% NP‐40, 0.5% Na deoxycholate, 0.1% SDS, 5 cOmplete™ protease inhibitor tablets) was added followed by sonication using 5 × 1 s pulses at 40% amplitude per embryo (High Intensity Ultrasonic Processor, SONICS VIBRA CELL). Embryo lysates were placed on ice, centrifuged at 10,000*g* for 20 min at 4 °C, and the DNA pellet was discarded. Protein content was determined using the bicinchoninic acid (BCA) assay (Smith et al., 1985). About 1 mL of each protein lysate and each BSA standard (0, 0.01, 0.02, 0.05, 0.1, 0.2, 0.5, and 1.0 mg/mL distilled water) were added to 20 μL of BCA solution (Pierce), prepared according to the manufacturer's protocol. Samples were incubated at 37 °C for 45 min, and absorbance read at 562 nM on a Thermofisher NanoDrop 1000 Spectrophotometer. Sample protein contents were determined from the BSA standard line.

### 2.7 Literature review

PubMed (NCBI) was searched for the term: “whole_embryo_culture,” or individual component words. “Postimplantation,” “rat,” and “mouse” were used to narrow the searches. While this strategy identified the majority of WEC papers, some studies lacking these search terms in their titles may have been missed. Titles and abstracts were read individually to eliminate culture studies using preimplantation embryos and to determine whether teratogen effects were the goal of each WEC study. Methods sections were read to determine details of culture medium used in each study and whether comparisons of culture success had been made.

### 2.8 Statistical analysis

One‐way analysis of variance was used to test for overall significance in parametric growth measurements: yolk sac diameter, dorsal length, head length, somite number, and protein content. Where significant, post hoc pair‐wise Student's *t* tests (adjusted for multiple comparisons by the Bonferroni method) were performed in comparisons of serum‐free or diluted media versus RS controls. Chi‐square tests were used to test for overall significance in yolk sac circulation score frequencies (3 × 4) and axial rotation score frequencies (3 × 3). Where significant, post hoc pairwise chi‐square tests (2 × 4 and 2 × 3) were performed to compare serum‐free or diluted media with RS controls.

## 3 RESULTS

Two serum‐free media have been reported in mouse WEC (Table S1). KnockOut DMEM plus KSR were the main medium constituents in studies beginning at E10.5 (Kalaskar & Lauderdale, 2014; Moore‐Scott, Gordon, Blackburn, Condie, & Manley, 2003). N2B27, a further serum‐free medium, was used for 24 and 48 hr cultures of E5.5 mouse embryos (Drakou & Georgiades, 2015). Moreover, several studies have used diluted RS as culture medium, although without formal comparison against 100% in‐house‐produced RS for efficacy (Table S1). We evaluated mouse embryo development at E8.5–9.5 in serum‐free media and in RS diluted with various defined media, compared with 100% RS controls. Embryonic assessment included postculture diameter and blood circulation of yolk sac, axial rotation, dorsal and head length, somite number, total protein content, and cranial neural tube closure.

### 3.1 Serum‐free media fail to replicate the development of embryos cultured in RS

**Figure 3:**
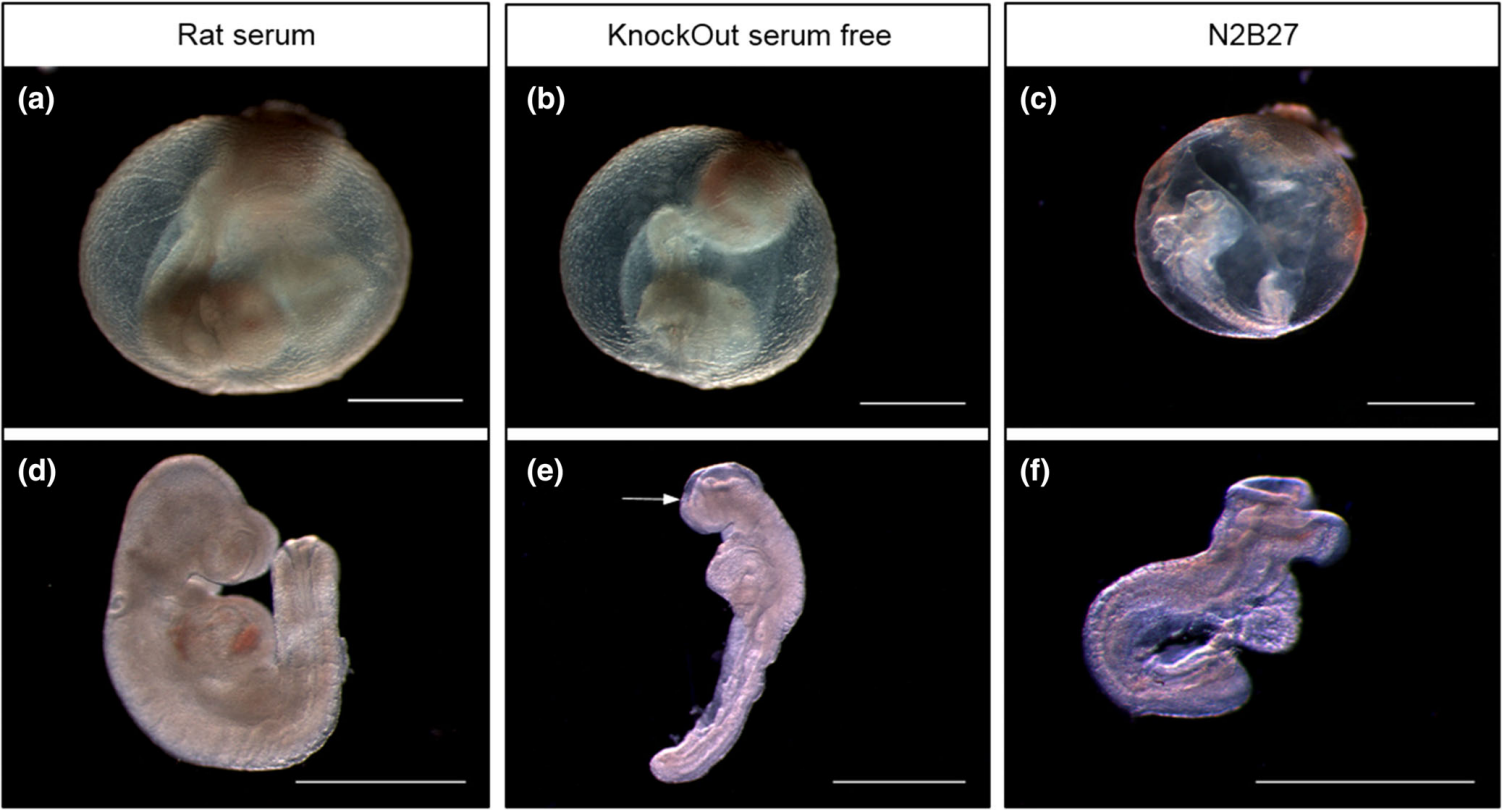
Comparison of whole embryo culture using rat serum (RS) or serum‐free media. Representative images of yolk sac (a–c) and isolated embryos (d–f) following 24 hr culture from E8.5 in RS (a and d), KnockOut serum‐free medium (b and e) or N2B27 serum‐free medium (c and f). (a and d) RS culture was generally associated with extensive vasculature and rapid blood movement in the yolk sac (e.g., score of 3 shown in a) together with fully turned embryos that exhibited a mostly normal appearance for this developmental stage. (b and e) KnockOut serum‐free culture produced conceptuses with evidence of yolk sac blood islands but little blood movement (score of 1 shown in b) and embryos that were growth retarded, incompletely turned, and with an open cranial neural tube (arrow in e). (c and f) N2B27 serum‐free culture yielded conceptuses with no evidence of yolk sac circulation (score of 0 shown in c) together with embryos that were unturned, growth retarded and with open cranial neural tube. Note that the embryo in (c) was completely unturned although it secondarily developed a convex dorsal surface following removal from the yolk sac (in f). Scale bars: 1 mm

**Figure 4:**
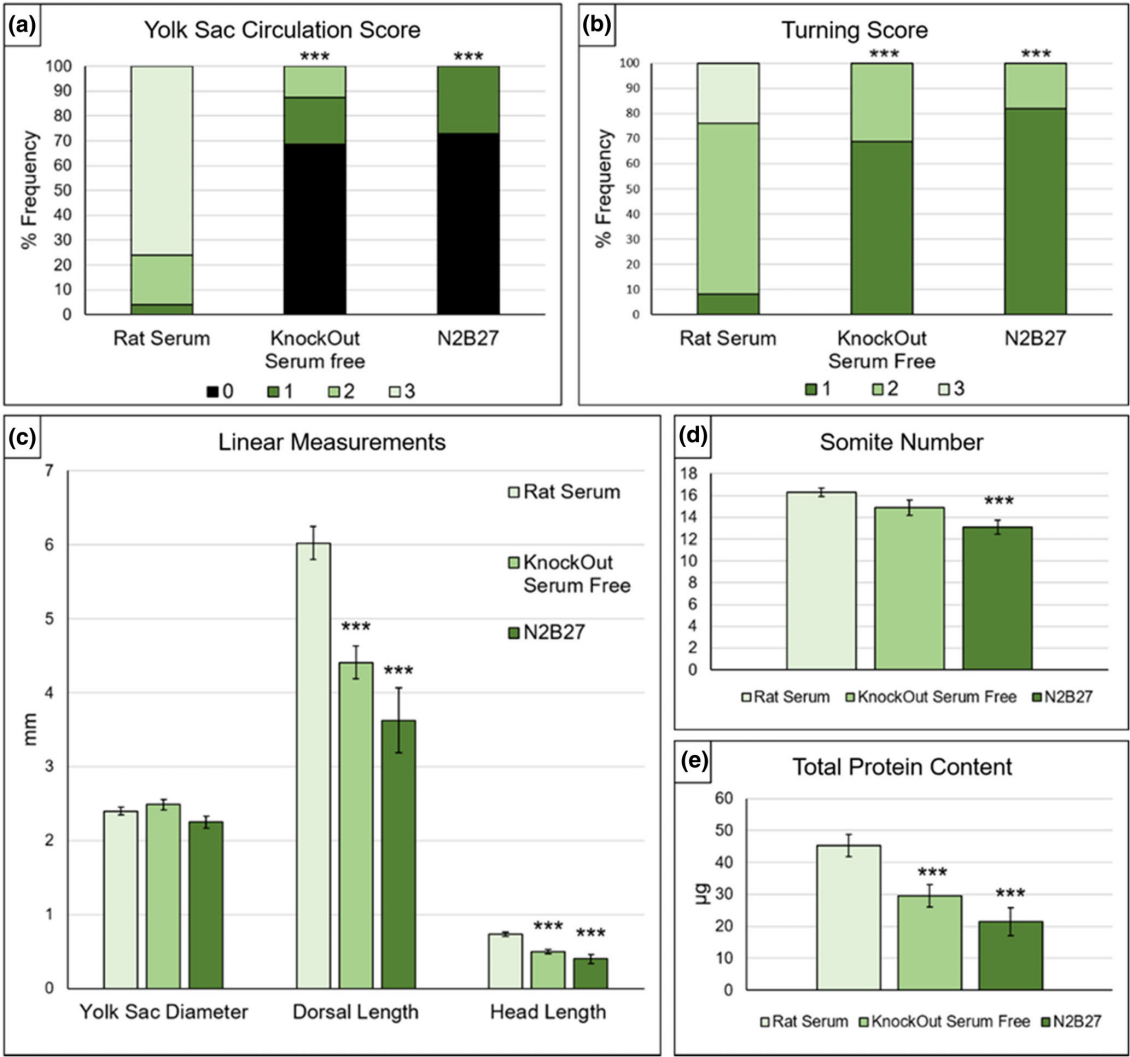
Developmental and growth parameters of embryos cultured in rat serum (RS) or serum‐free media. (a) Yolk sac circulation scores. KnockOut serum‐free (KOSF) medium and N2B27 produce significantly lower scores than culture in RS. (b) Turning scores. KOSF medium and N2B27 embryos have significantly lower scores than RS‐cultured embryos. (c) Yolk sac and embryo size measurements (mean ± *SEM*). RS and serum‐free media do not differ significantly in yolk sac diameter, whereas both dorsal length and head length are significantly reduced after culture in serum‐free media versus RS. (d) Somite numbers. Embryos cultured in KOSF medium show no significant difference from RS controls, whereas embryos cultured in N2B27 have significantly fewer somites. (e) Total protein content. Embryos from both serum‐free media cultures have significantly reduced total protein content compared with RS controls. Statistical analysis: (a and b) Chi‐square tests comparing all score categories across the 3‐culture media are significant for both yolk sac circulation and turning (*p* ≤ .001). Post hoc tests of serum‐free media versus RS: ***p ≤ .001; 2 × 4 chi‐square test for yolk sac circulation and 2 × 3 chi‐square test for turning score. (c–e) One‐way ANOVA shows significant variation in each measurement between the 3‐culture media (*p* ≤ .01), with the exception of yolk sac diameter. Post hoc tests of serum‐free media versus RS: ***p ≤ .001, Student's *t* tests. Rat serum: *n* = 25; KnockOut serum‐free medium: *n* = 16; N2B27 serum‐free medium: *n* = 11

Yolk sac circulation is an important indicator of embryo health in culture (Pinter et al., 1986). Embryos cultured in KnockOut serum‐free medium showed poor yolk sac development, with blood islands but little evidence of circulation and no mature developed blood vessels, unlike embryos cultured in whole RS (Figure 3a,b). The majority of KnockOut serum‐free embryos had a yolk sac score of zero (69%), whereas 76% of littermates cultured in RS achieved a maximum score of 3. Among embryos cultured in serum‐free N2B27, 73% scored zero with most showing no evidence of blood islands (Figure 3c) and none having a yolk sac blood circulation. Both serum‐free media had significantly lower yolk sac circulation scores than RS controls (Figure 4a). In contrast, yolk sac diameter showed no significant difference between the three culture conditions (Figure 4c).

Axial rotation is another important developmental landmark that embryos should achieve when cultured from the head‐fold stage (Matsuda & Yasutomi, 1995). Of embryos grown in RS, 24% had fully turned (score of 3) and 68% were in the process of turning (score of 2) at the end of 24 hr culture (Figures 3a,d and 4b). In contrast, none of the embryos in either serum‐free medium had fully turned. Only 31% of embryos cultured in KnockOut medium were turning and 69% were entirely unturned (Figures 3b and 4b) whereas, for N2B27 medium, 18% were partially turned and 82% were unturned (Figures 3c and 4b). These results for serum‐free media were significantly different from RS‐cultured embryos.

In terms of growth parameters, both dorsal length and head length were significantly reduced in serum‐free media compared with RS cultures. Mean (±*SEM*) dorsal length in RS culture was 6.02 ± 0.22 mm, whereas embryos in serum‐free medium had values of 4.41 ± 0.22 mm (KnockOut medium) and 3.63 ± 0.43 mm (N2B27). Head length was 0.73 ± 0.03 mm in RS, compared with 0.49 ± 0.03 mm in KnockOut medium, and 0.40 ± 0.06 mm in N2B27 medium (Figure 4c).

Somite number shows a linear increase with time between E8.5 and E9.5, with a new somite pair added approximately every 2 hr. Hence, somite number is often used as a measure of developmental progression in embryo culture (Kalaskar & Lauderdale, 2014). E8.5 CD‐1 embryos prior to culture typically had 3–6 somites suggesting that 24 hr later these embryos should exhibit 15–19 somites. Mean somite number in RS‐cultured embryos (16.3 ± 0.39) fell within this expected range, whereas embryos cultured in serum‐free media had fewer somites. While somite number in KnockOut serum‐free cultures (14.9 ± 0.71) did not differ significantly from RS controls, culture in N2B27 yielded a significantly lower mean somite number (13.1 ± 0.64; Figure 4d).

Failure of cranial neural tube closure leads to exencephaly, a neural tube defect that commonly affects the midbrain and hindbrain of mouse embryos. The frequencies of open cranial neural tube in this study were: 2/25 (open/total) in RS, 7/16 in KnockOut medium and 9/11 in N2B27 medium. It is important to note, however, that the mid/hindbrain of most mouse strains remains open until the 16 somite stage (MacDonald, Juriloff, & Harris, 1989), so exencephaly can only be conclusively identified in embryos with ≥17 somites. While 12/25 embryos cultured in RS had ≥17 somites, only one embryo in KnockOut medium and no embryos in N2B27 reached this somite stage. Hence, the higher apparent rate of cranial closure failure in the serum‐free media cultures may be related to the relative developmental retardation of the embryos.

Protein content was measured in embryo lysates, to assess total embryonic growth (Figure 4e). Embryos cultured in RS had a mean (±*SEM*) protein content of 45.2 ± 3.53 μg; whereas embryos in serum‐free media contained significantly less protein: 29.6 ± 3.50 μg (KnockOut) and 21.5 ± 4.38 μg (N2B27).

In conclusion, taking all the developmental parameters together, neither of the serum‐free media satisfactorily supported embryonic development in a comparable way to 100% RS.

### 3.2 Diluted RS can support normal embryonic development in culture

Four different serum‐free media were tested as diluents for RS, to evaluate the prospects of reducing serum usage in embryo culture. KnockOut serum‐free medium, N2B27 medium, DMEM and GMEM + DS were each used to dilute RS 1:1, with evaluation of growth and development after 24 hr culture from E8.5.

**Figure 5:**
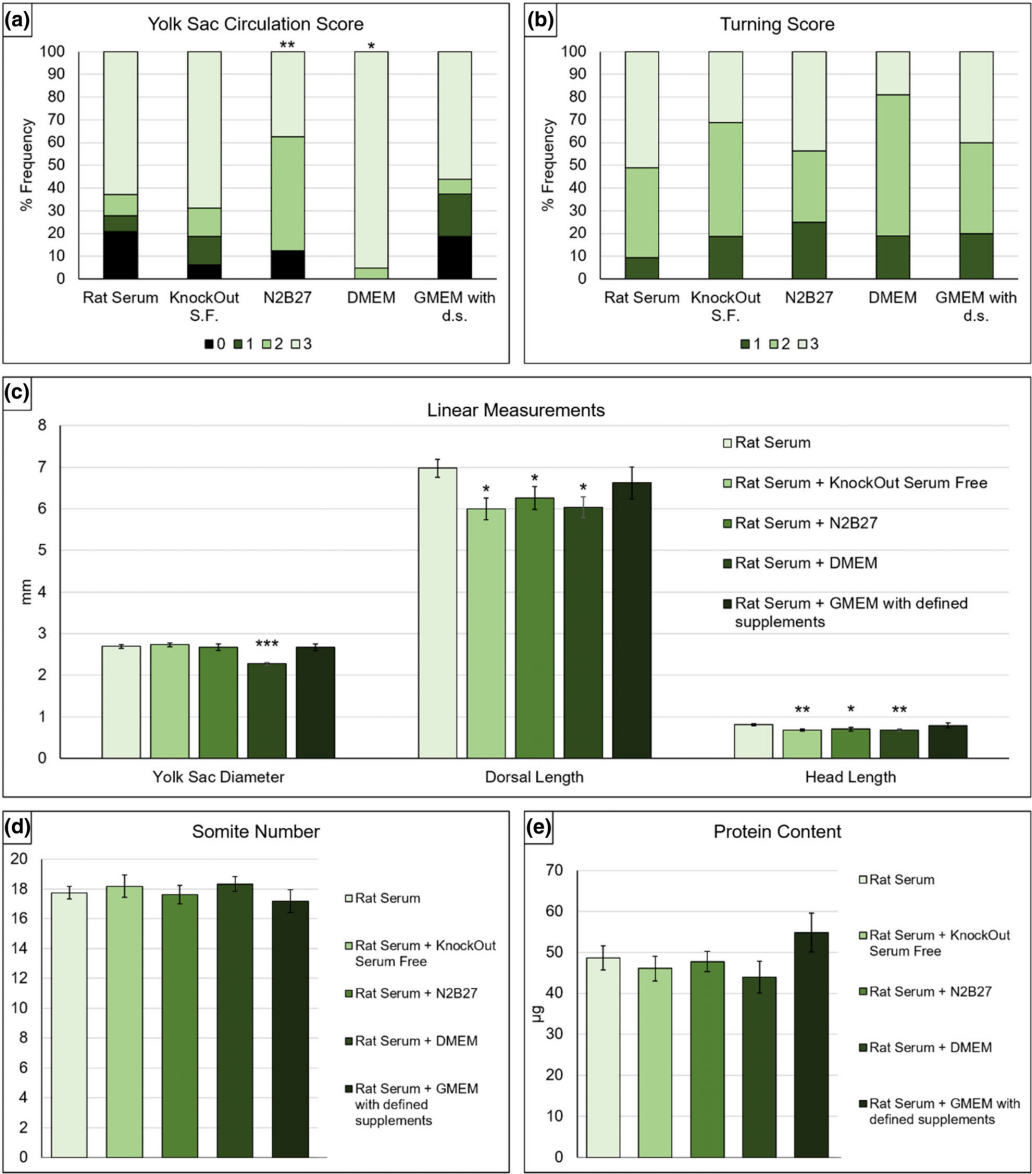
Developmental and growth parameters of embryos cultured in rat serum (RS) or in the four combination (diluted) media. (a) Yolk sac circulation scores. RS diluted with N2B27 supports significantly diminished yolk sac circulation compared with 100% RS, whereas dilution with DMEM produces significantly enhanced scores. Dilution with KnockOut serum‐free (KOSF) medium or GMEM + defined supplements (DS) shows no significant difference from 100% RS controls. (b) Turning score. No significant differences in turning score are observed between 100% RS and the four combination media. (c) Yolk sac diameter is significantly reduced when DMEM is the diluent, whereas other media do not differ from the RS control. Dorsal length is significantly reduced in cultures containing KOSF medium or DMEM. Head length is significantly reduced by all diluents except GMEM + DS, which does not differ from 100% RS. (d) Somite number. No significant differences are observed between 100% RS and the four combination media. (E) Total protein content. No significant differences in turning score are observed between 100% RS and the four combination media. Statistical analysis: (a and b) Chi‐square tests comparing all score categories across the five culture media are significant for yolk sac circulation (*p* ≤ .001) but not for turning score (*p* > .05). Post hoc tests of combination media versus RS: **p ≤ .01 and *p ≤ .05; 2 × 4 chi‐square tests for yolk sac circulation. (c–e) One‐way ANOVA shows significant variation in growth measurement between the five culture media (*p* ≤ .05) but not in somite number or protein content. Post hoc tests of combination media versus RS: ***p ≤ .001, **p ≤ .01, *p ≤ .05; Student's *t* tests. RS: *n* = 43. Diluted media: KOSF: *n* = 16; N2B27 serum‐free: *n* = 16; DMEM: *n* = 21; GMEM + DS: *n* = 16

Yolk sac circulation achieved a maximum score of 3 in 50% or more of embryos cultured in 100% RS and in all of the diluted media with the exception of N2B27, in which fewer than 40% embryos achieved a score of 3, significantly lower than in 100% RS (Figure 5a). The proportion with a score of 3 was significantly higher (95%) in RS diluted with DMEM compared with 100% RS. All diluted media gave markedly higher yolk sac scores than the two serum‐free media when used in the absence of RS (Figure 4a). In contrast to yolk sac score, axial rotation (“turning”) scores in all combination media did not differ from undiluted RS (Figure 5b). Yolk sac diameter was significantly reduced when RS was diluted with DMEM; whereas other combination media did not differ from 100% RS (Figure 5c).

In terms of embryo growth and developmental progression, dorsal length and head length were both reduced significantly in all combination media except when GMEM + DS was used as diluent, in which case there was no difference from 100% RS (Figure 5c). There were no differences in either somite number (Figure 5d) or total protein content (Figure 5e) between embryos cultured in undiluted RS and any of the diluted media. The apparently increased protein content in GMEM + DS was not significantly different from RS controls.

**Figure 6:**
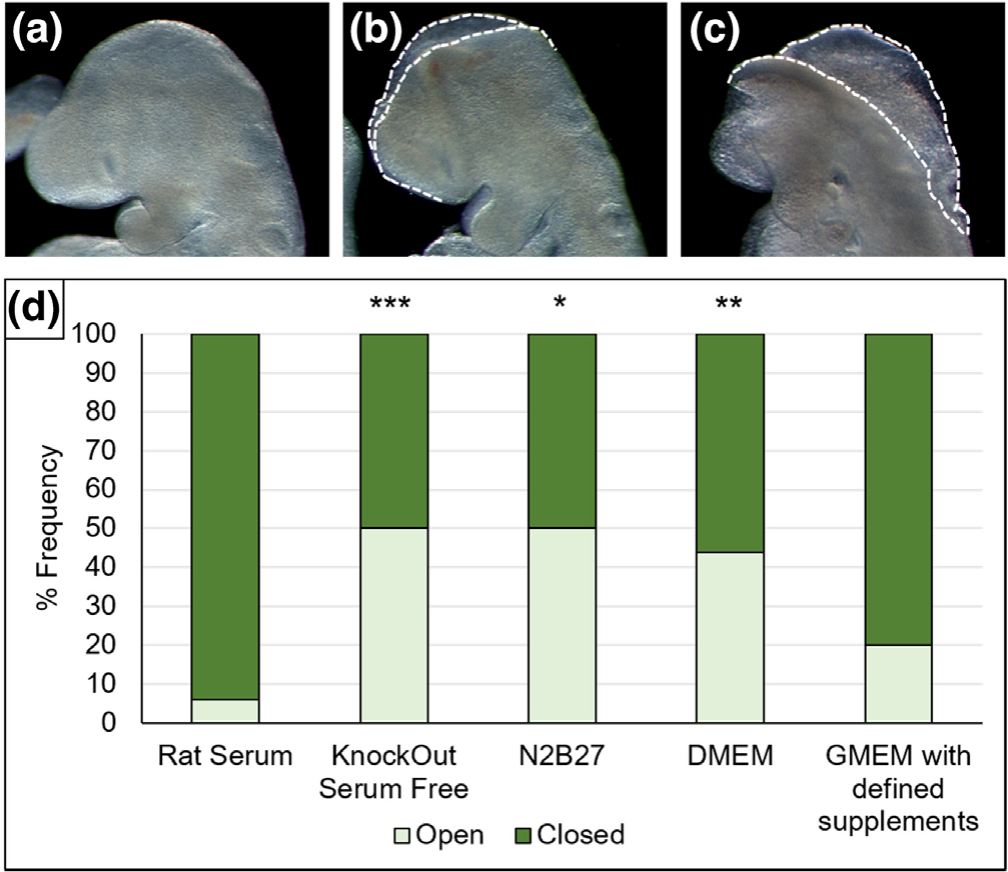
Frequency of open cranial neural tube in embryos cultured in rat serum (RS) or in the four combination (diluted) media. (a–c) Example images to demonstrate the range of cranial neural tube closure phenotypes observed. (a) Closed cranial neural tube. (b) Open cranial neural tube of minor severity which is likely to complete closure during subsequent development. (c) Open cranial neural tube of major severity, with widely spaced, everted neural folds extending into the hindbrain region. Embryos with this appearance are unlikely to complete cranial neural tube closure. (d) Dilution of RS with KnockOut serum‐free (KOSF) medium, N2B27 medium and DMEM are all associated with a significant increase in the proportion of embryos with ≥17 somites that have an open cranial neural tube. GMEM + DS shows no significant differences, compared with 100% RS. Statistical analysis: Chi‐square test comparing frequency of open cranial neural tube across the five culture media is significant (*p* ≤ .05). Post hoc tests of combination media versus RS: ***p ≤ .001, **p ≤ .01, *p ≤ .05; 2 × 2 chi‐square tests. Numbers of embryos with ≥17 somites: RS: *n* = 33; KOSF medium: *n* = 12; N2B27 serum‐free medium: *n* = 12; DMEM: *n* = 17; GMEM + DS: *n* = 10

Cranial neural tube closure was completed in 31/33 embryos with ≥17 somites cultured in 100% RS (Figure 6a,d). Of the two with incomplete cranial closure, the brains appeared almost closed (as in Figure 6b). Similarly, the majority (8/10) of embryos with ≥17 somites cultured in RS diluted with GMEM + DS had a closed brain, not significantly different from RS controls (Figure 6d). In contrast, KnockOut medium, N2B27 and DMEM diluents all yielded a significantly higher proportion of open midbrain in embryos with ≥17 somites, compared with undiluted RS. Moreover, 50–100% of these embryos exhibited widely splayed neural folds, suggesting incipient exencephaly (as in Figure 6c).

Throughout these dilution experiments, a volume of 0.5–1 mL of medium was used per embryo in all treatment groups. However, typical mouse culture studies include three embryos per milliliter of medium from E8.5 to E9.5. To assess whether a smaller medium volume might compromise any of the developmental parameters, five embryos were cultured in 1.5 mL of RS diluted with GMEM + DS. No significant changes were seen in any of the growth or developmental parameters compared with the larger volume cultures (data not shown).

In summary, GMEM + DS is the only diluent that shows no statistically significant difference in any growth or developmental parameter, when compared with 100% RS (Figures 5 and 6). All other diluents show statistically significant reductions in one or more of the parameters. We conclude that it is possible to reproduce embryonic growth and development to the quality of 100% RS using GMEM + DS as a 50% diluent.

### 3.3 RS dilution of more than 50% results in suboptimal embryo growth in culture

**Figure 7:**
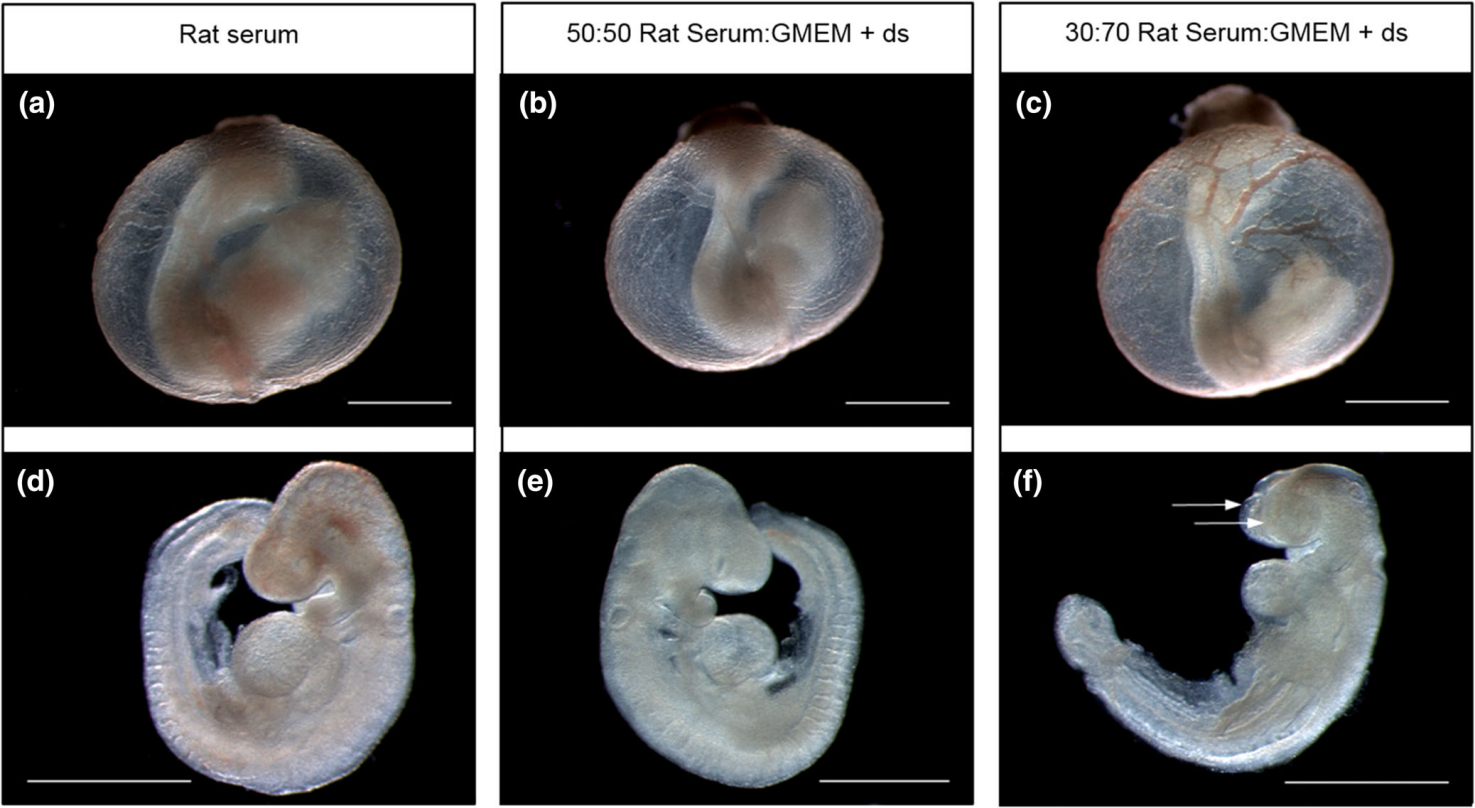
Effect of increased dilution of rat serum (RS) on success of embryo culture. Representative images of yolk sacs (a–c) and isolated embryos (d–f) cultured in 100% RS (a and d), or in RS diluted 50:50 (b and e) or 30:70 (c and f) with GMEM plus defined supplements (ds). (a–c) Yolk sac development is closely comparable in 100% RS and in both dilutions with GMEM + ds. All three examples (a–c) scored a maximum of 3 on the yolk sac circulation scale. (d–f) Morphological appearance of embryos is closely similar in 100% RS (d) and in serum diluted 50:50 with GMEM + ds (e). Embryos are fully turned, of similar size, and with no obvious abnormalities. In contrast, embryos cultured in RS diluted 30:70 with GMEM + ds (f) exhibit reduction in growth parameters (e.g., small head) and more often have an open cranial neural tube (arrows). Scale bars: 1 mm

Greater dilution of RS, for example to 30% of total volume, would represent a further saving in rat usage for embryo culture. Embryos were cultured in RS diluted 30:70 with GMEM + DS as the diluent, by comparison with 100% RS controls. Embryos appeared healthy in the 30:70 diluted medium: for example, 10/11 embryos achieved a yolk sac score of 3, indicating extensive vasculature and rapid blood circulation in most cases (Figure 7). This proportion did not differ significantly from parallel RS control cultures (5/7 with yolk sac score of 3). However, in terms of neural tube development, 2/3 embryos with ≥17 somites in the 30:70 diluted medium exhibited an open cranial neural tube (Figure 7f), in comparison to only 1/4 embryos (and 2/33 in Figure 6d) with ≥17 somites in 100% RS controls, where the brain appeared almost closed (as in Figure 6b).

**Figure 8:**
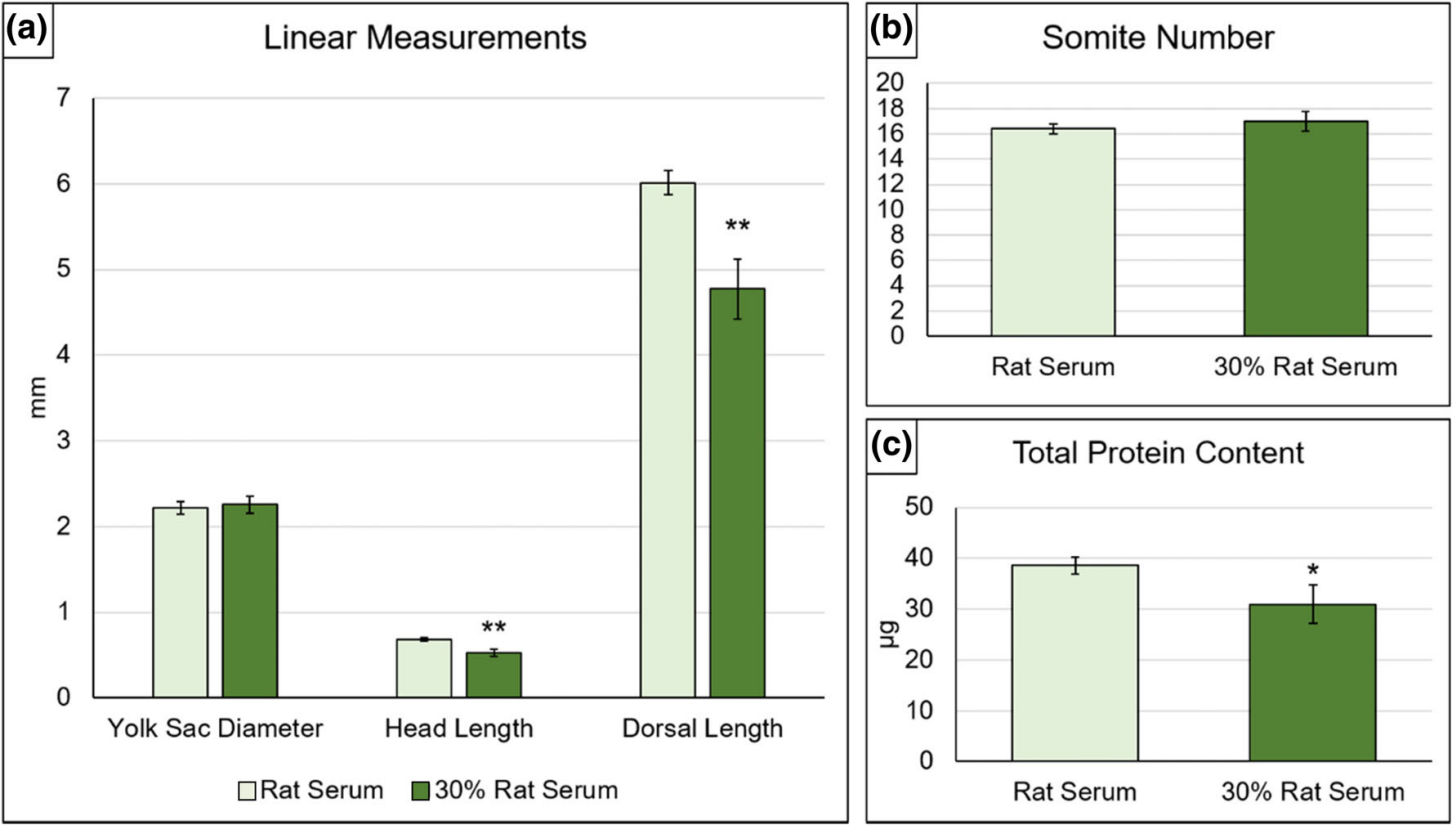
Developmental and growth parameters of embryos cultured in 100% rat serum (RS) or in serum diluted 30:70 with GMEM plus DS. (a) Yolk sac diameter shows no difference between 100% and diluted RS cultures, whereas dorsal length and head length are both significantly diminished in the 30% RS cultures. (b) Somite number does not differ significantly in embryos cultured in 100% or 30% RS. (c) Total protein content shows a significant reduction in 30% RS compared with 100% RS controls. Statistical analysis: **p ≤ .01, *p ≤ .05; Student's *t* tests. RS: *n* = 7, 30% RS: *n* = 11

Neither yolk sac diameter (Figure 8a), axial rotation score (data not shown) nor somite number (Figure 8b) showed significant differences between the 30:70 diluted medium and 100% RS. In contrast, growth parameters were adversely affected: Mean dorsal length and head length (Figure 8a) and total protein content (Figure 8c) were significantly smaller in 30:70 diluted medium compared with 100% RS.

Hence, 70% dilution of RS by GMEM + DS is unable to replicate development in 100% RS culture. Although embryo health and developmental progression are comparable, there is a significant adverse effect on embryonic growth parameters and there appears to be a higher incidence of cranial neural tube closure delay or failure. This finding would preclude the use of RS diluted by more than 50% for experimental studies in the E8.5‐E9.5 embryonic period.

### 3.4 Addition of glucose does not restore development in 30% RS‐cultured embryos

Previous studies of rat embryos undergoing organogenesis in vitro found that addition of glucose could restore embryo growth in a minimal medium (Cockroft, 1979). To investigate a possible effect of glucose addition in the current study, E8.5 embryos were cultured for 24 hr in the 30:70 dilution of RS by GMEM + DS, with the addition of 2 mg/mL glucose. Although some improvement in growth parameters was seen, yolk sac diameter (*p* ≤ .01) and head length (*p* ≤ .05) both showed significant reductions compared with 100% RS cultures (data not shown). Hence, the addition of glucose is unable to rescue the compromised growth of mouse embryos cultured in RS diluted 70% with serum‐free medium.

## 4 DISCUSSION

Previous WEC studies with both rat and mouse embryos identified approximately parallel progression of development in utero and in embryos cultured in vitro using RS (New, Coppola, & Cockroft, 1976; Sadler, 1979). This finding forms an important rationale for using WEC in research studies of normal embryonic development and the origin of congenital defects. Indeed, our literature survey confirmed that mouse WEC in particular continues to be used widely for a variety of developmental biology studies, with related technologies including gene transfer and live imaging being adapted for use with WEC.

Evidence‐based changes in experimental protocols are essential for the progressive improvement of commonly used methodologies. The early research with rat WEC (New, 1978) involved rigorous comparisons of culture methodology, which defined several critical aspects of the WEC procedure. Immediate centrifugation of rat blood during serum preparation (Steele & New, 1974), a gas atmosphere transitioning with developmental stage from hypoxic (5% O_2_) to hyperoxic (40–95% O_2_) (New & Coppola, 1970a; New & Coppola, 1970b), and continuous mixing of the medium with gas atmosphere: For example, circulators, rotators or roller bottles (New, 1967; New et al., 1973; New & Cockroft, 1979) were all demonstrated to be necessary for achieving development in culture comparable to in vivo.

The choice of culture medium is a further critical aspect of the WEC procedure and, since the early studies, a variety of media have been used in WEC. These include alternative sera to RS, dilution of RS to differing extents with various defined media, and a few serum‐free media (Table S1). Strikingly, however, almost no formal comparisons have been reported of variant WEC media against the “gold standard” of 100% RS. For example, authors reporting “successful” cultures using serum‐free media (Drakou & Georgiades, 2015; Kalaskar & Lauderdale, 2014), did not include side‐by‐side comparisons with RS‐based medium. We attempted to resolve this issue by performing a formal comparison of serum‐free and diluted RS‐based media against 100% RS. Limitations of our study include testing of only 24 hr cultures from E8.5 (chosen as a particularly popular culture period for mouse WEC) and the use of a single random‐bred strain of mice (chosen as representative of many random‐bred strains used in WEC). Given these limitations, we demonstrate that use of RS cannot be replaced by serum‐free medium in cultures from E8.5, without serious loss of embryo quality. Nevertheless, it is possible to dilute RS 1:1 with GMEM + DS, but not other media including DMEM, and still achieve embryonic growth and development comparable to 100% RS.

While 50% dilution of RS supported development as well as undiluted RS, greater dilution by 70% produced suboptimal embryonic growth. It would be interesting to determine the factors in RS that are dilution‐limiting. Early experiments studying the components of RS that change over the culture period found the most significant decrease was in glucose concentration, whose exhaustion coincided with embryonic death (Sanyal, 1980). It was also shown in experiments with dialyzed serum, that the addition of glucose as an energy source, restored growth to control levels (Cockroft, 1979). We added glucose to the 70% diluted RS medium but growth parameters remained significantly impaired, compared with 100% RS control embryos. Vitamins including pantothenic acid, riboflavin, and inositol are essential constituents of RS as a culture medium, at certain concentrations (Cockroft, 1979). Further experiments involving these vitamins and perhaps other growth factors could potentially enable the RS component of embryo culture medium to be reduced below 50%.

Our findings raise the question of why GMEM + DS supports better embryonic growth than other media, including DMEM, when used as a diluent for RS. GMEM + DS and DMEM contain a similar range of inorganic salts, amino‐acids, and vitamins (Sigma, datasheets) although GMEM + DS contains half the concentration of most amino‐acids and vitamins compared with DMEM. However, the non‐essential amino acids (NEAAs): alanine, asparagine, aspartic acid, and proline are present in GMEM + DS but not in DMEM, while the concentration of L‐glutamine is also greater in GMEM + DS than DMEM (Table S2).

Early mammalian embryos show precise requirements for amino‐acids in culture medium. At the preimplantation stage, NEAAs and L‐glutamine were found to enhance both cleavage divisions to the 8‐cell stage and formation of the blastocyst (Lane & Gardner, 1997; Van Winkle, 2001). Moreover, after transfer to the uterus, cultured preimplantation embryos yielded the highest rates of implantation and postimplantation development when all 20 amino‐acids (11 nonessential + 9 essential) were included in the culture medium (Lane & Gardner, 1997). Amino‐acids are also incorporated into embryonic protein during rat WEC, although the predominant source of new protein synthesis is from breakdown of serum proteins by the yolk sac (Beckman, Brent, & Lloyd, 1996). Hence, it is possible that the presence of additional NEAAs in GMEM + DS contributes to its superior performance as a diluent for RS.

RS reduction by 50% could represent a significant step toward realizing the goals of the National Centre for the 3Rs (NC3Rs), which aims to replace, reduce, and refine animal use in research (Burden, Chapman, Sewell, & Robinson, 2015). WEC, as it stands, provides a refinement of developmental studies on rodent embryos, as it allows multiple treatments and conditions to be applied to the same litter from a single pregnant dam, ensuring a lower number of dams are exposed to harmful procedures or sacrificed. A move toward reducing amounts of RS used in WEC would represent a further progression along the route toward minimizing animal usage in developmental biology research.

In conclusion, the present study provides an evidence‐base for mouse embryo cultures using diluted RS, and demonstrates that mouse WEC cannot currently be performed successfully in a serum‐free medium.

## CONFLICT OF INTERESTS

Andrew Copp acts as paid consultant for ViiV Healthcare Limited, with fees going to support his research program. The other authors declare no conflicts of interest.

## AUTHOR CONTRIBUTIONS

Research concept and funding: A.J.C., N.D.E.G., and D.S. Research design: L.H.C. and A.J.C. Experiments and data analysis: L.H.C. Animal procedures: L.H.C. and D.S. Manuscript preparation: L.H.C. and A.J.C. Manuscript editing: L.H.C., D.S., N.D.E.G., and A.J.C.

## Supporting information


**Table S1** Literature review of mouse embryo culture studies using variations from 100% rat serum (RS^*^) as culture medium

**Table S2** Comparison of the amino‐acid compositions of DMEM and GMEM + defined supplements (DS), as used to dilute rat serum for whole embryo culture

## Data Availability

The data that support the findings of this study will be made openly available in a recognized repository from the time of article publication.
