# Supplementary material for: Mouse whole embryo culture: Evaluating the requirement for rat serum as culture medium
Source: Birth Defects Res. 2019 Jun 24;111(16):1165–77. doi: 10.1002/bdr2.1538 (PMC6778057; doi:10.1002/bdr2.1538)
Supplement: Supplementary file 1 — Table S1 Literature review of mouse embryo culture studies using variations from 100% rat serum (RS*) as culture medium [file BDR2-111-1165-s001.docx]

**Table S1. Literature review of mouse embryo culture studies using variations from 100% rat serum (RS *) as culture medium**

|  | **Paper** | **Stages cultured** | **Brief culture period  < 18 h?** | **Culture medium variations** | | | **Comparisons of culture success (excluding effects of experimental treatments)** |
| --- | --- | --- | --- | --- | --- | --- | --- |
|  |  |  |  | **Serum- free** | **Rat serum dilution** | **Non-rat serum** |  |
|  | Miyazawa H., Yamamoto M., Yamaguchi Y., and Miura M. (2018). Mammalian embryos show metabolic plasticity toward the surrounding environment during neural tube closure**.** *Genes Cells* **23:** 794-802. | E7.5; E8.5; E9.5 | Yes (1.5 h) |  | RS 1:1 with OPTI‐MEM |  | No |
|  | Ivanovitch K., Temino S., and Torres M. (2017). Live imaging of heart tube development in mouse reveals alternating phases of cardiac differentiation and morphogenesis. *Elife* **6**: e30668. | E7.5 | Yes (3-13 h) |  | RS 1:1 with DMEM + 1% N-2 neuronal growth suppl + 1% B-27 suppl |  | No |
|  | Huang Y., Wilkie R., and Wilson V. (2015). Methods for precisely localized transfer of cells or DNA into early postimplantation mouse embryos**.** *J. Vis. Exp.* **106:** e53295. | E7.5; E8.5; E9.5 | No |  | RS 1:1 or 1:3 with Glasgow (G)MEM, 1% Non-Essential Amino Acids, L-glutamine, Na pyruvate |  | No |
|  | Drakou K. and Georgiades P. (2015). A serum-free and defined medium for the culture of mammalian postimplantation embryos**.** *Biochem. Biophys. Res. Commun.* **468:** 813-819. | E5.5-E7.5 | No | N2B27: serum-free medium |  |  | Yes - against in vivo embryo staging system |
|  | Kimura-Yoshida C., Mochida K., Ellwanger K., Niehrs C., and Matsuo I. (2015). Fate specification of neural plate border by canonical Wnt signaling and Grhl3 is crucial for neural tube closure. *EBioMedicine.* **2:** 513-527. | E8.5-E9.0 | Yes (6 h) |  |  | FBS 1:4 with DMEM | No |
|  | Kalaskar V.K. and Lauderdale J.D. (2014). Mouse embryonic development in a serum-free whole embryo culture system**.** *J. Vis. Exp.* **85:** e50803. | E10.5-E11.5 | No | Knockout serum-free medium |  |  | Yes - against in vivo developed embryos |
|  | Gordon J., Moore B.A., Blackburn C.C., and Manley N.R. (2014). Serum-free culture of mid-gestation mouse embryos: a tool for the study of endoderm-derived organs**.** *Methods Mol. Biol.* **1092:** 183-194. | E10.5-E11.5 | No | Knockout serum- free medium |  |  | No |
|  | Glanville-Jones H.C., Woo N., and Arkell R.M. (2013). Successful whole embryo culture with commercially available reagents**.** *Int. J Dev. Biol.* **57:** 61-67. | E7.5-E8.5; E8.5-E9.5 | No |  | RS (commercial **) 1:1 with DMEM or Ham's F12 with N-2 growth supplement |  | Yes - between two diluent media |
|  | Mahaffey J.P., Grego-Bessa J., Liem K.F., Jr., and Anderson K.V. (2013). Cofilin and Vangl2 cooperate in the initiation of planar cell polarity in the mouse embryo**.** *Development* **140:** 1262-1271. | E7.25-E8.0 | No |  | RS 1:1 with DMEM/F12 |  | No |
|  | Zeeb M., Axnick J., Planas-Paz L., Hartmann T., Strilic B., and Lammert E. (2012). Pharmacological manipulation of blood and lymphatic vascularization in ex vivo-cultured mouse embryos. *Nat. Protoc.* **7:** 1970-1982. | E8.0; E8.75; E11.5; E15.5 | Yes (12 h, E8.0; 8 h, E8.75; 5 h, E11.5; 0.5 h, E15.5) | M16 + HEPES for E8.0 and E8.75 |  | FBS 1:4 with DMEM for E11.5 and E15.5 | No |
|  | Trichas G., Smith A.M., White N., Wilkins V., Watanabe T., Moore A., Joyce B., Sugnaseelan J., Rodriguez T.A., Kay D. et al. (2012). Multi-cellular rosettes in the mouse visceral endoderm facilitate the ordered migration of anterior visceral endoderm cells**.** *PLoS. Biol.* **10:** e1001256. | E5.75 | Yes (8 h) |  |  | MS 1:1 with CMRL containing glutamine | No |
|  | Shinohara K., Kawasumi A., Takamatsu A., Yoshiba S., Botilde Y., Motoyama N., Reith W., Durand B., Shiratori H., and Hamada H. (2012). Two rotating cilia in the node cavity are sufficient to break left-right symmetry in the mouse embryo**.** *Nat. Commun.* **3:** 622. | E8.5-9.5 | No |  | RS 3:1 with DMEM |  | No |
|  | Gray J. and Ross M.E. (2011). Neural tube closure in mouse whole embryo culture**.** *J. Vis. Exp.* **56:** e3132. | E8.5-10 | No |  | RS 1:1 with DMEM |  | No |
|  | Tung E.W. and Winn L.M. (2011). Valproic acid increases formation of reactive oxygen species and induces apoptosis in postimplantation embryos: a role for oxidative stress in valproic acid-induced neural tube defects**.** *Mol. Pharmacol.* **80:** 979-987. | E8.5-9.5 | No |  | RS 9:1 with Hanks' balanced salt solution (HBSS) |  | No |
|  | Mao J., McKean D.M., Warrier S., Corbin J.G., Niswander L., and Zohn I.E. (2010). The iron exporter ferroportin 1 is essential for development of the mouse embryo, forebrain patterning and neural tube closure**.** *Development* **137:** 3079-3088. | E7.5-8.5 | No |  | RS 1:1 with DMEM +HEPES, pen/strep |  | No |
|  | Migeotte I., Omelchenko T., Hall A., and Anderson K.V. (2010). Rac1-dependent collective cell migration is required for specification of the anterior-posterior body axis of the mouse**.** *PLoS. Biol.* **8:** e1000442. | E5.5-E6.5 | No |  | RS 1:1 DMEM/F12 |  | No |
|  | Pyrgaki C., Trainor P., Hadjantonakis A.K., and Niswander L. (2010). Dynamic imaging of mammalian neural tube closure**.** *Dev. Biol* **344:** 941-947. | E8.5-9.5 | No |  | RS 2:1 DMEM/F12 |  | No |
|  | Miura S. and Mishina Y. (2003). Whole-embryo culture of E5.5 mouse embryos: Development to the gastrulation stage**.** *Genesis* **37:** 38-43. | E5.5-E7.5 | No |  | RS 100%, 3:1 or 1:1 with DMEM |  | Yes - between 50%, 75% and 100% RS |
|  | Soleman D., Cornel L., Little S.A., and Mirkes P.E. (2003). Teratogen-induced activation of the mitochondrial apoptotic pathway in the yolk sac of day 9 mouse embryos**.** *Birth Defects Research (Part A)* **67:** 98-107. | E8.5-E9 | Yes (5 h and 10 h) |  | RS 4:1 with HBSS |  | No |
|  | Torchinsky A., Brokhman I., Shepshelovich J., Orenstein H., Savion S., Zaslavsky Z., Koifman M., Dierenfeld H., Fein A., and Toder V. (2003). Increased TNF-alpha expression in cultured mouse embryos exposed to teratogenic concentrations of glucose**.** *Reproduction.* **125:** 527-534. | E8.5-E9.5 | No |  |  | FBS 3:1 with DMEM + pen/ strep | Yes - against in vivo developed embryos |
|  | Moore-Scott B.A., Gordon J., Blackburn C.C., Condie B.G., and Manley N.R. (2003). New serum-free in vitro culture technique for midgestation mouse embryos**.** *Genesis* **35:** 164-168. | E10.5-11.5 | No | Knock Out DMEM + 10–20% KSR |  |  | Yes - against in vivo developed embryos |
|  | Jones E.A., Crotty D., Kulesa P.M., Waters C.W., Baron M.H., Fraser S.E., and Dickinson M.E. (2002). Dynamic in vivo imaging of postimplantation mammalian embryos using whole embryo culture**.** *Genesis* **34:** 228-235. | E8.5-9.5; E9.5-10.5 | No |  | RS 1:1 with DMEM +HEPES, pen/strep |  | Yes - against in vivo embryos and staging system |
|  | Nonaka S., Shiratori H., Saijoh Y., and Hamada H. (2002). Determination of left-right patterning of the mouse embryo by artificial nodal flow. *Nature* **418***:* 96-99. | E7.5-E9.5 | No |  | RS 1:1 with DMEM + pen/strep |  | No |
|  | Penkov L.I., Platonov E.S., and New D.A. (1995). Prolonged development of normal and parthenogenetic postimplantation mouse embryos in vitro**.** *Int. J Dev. Biol.* **39:** 985-991. | E8.5-E10.5 | No |  | RS 3:1 with DMEM + pen/strep |  | No |
|  | Van Maele-Fabry G., Gofflot F., and Picard J.J. (1995). Whole embryo culture of presomitic mouse embryos**.** *Toxicol. In Vitro* **9:** 671-675. | E7.5-E9.5; E8.5-E10.5 | No |  |  | MS: RS: HS 1:2:5 (1-24 h) then RS: HS 1:4 (25-48 h) | Yes - between different pre-somite stages at start of culture |
|  | Van Maele-Fabry G., Picard J.J., Attenon P., Berthet P., Delhaise F., Govers M.J., Peters P.W., Piersma A.H., Schmid B.P., Stadler J. et al. (1991). Interlaboratory evaluation of three culture media for postimplantation rodent embryos**.** *Reprod. Toxicol.* **5:** 417-426. | E8.5-E10.5 | No |  |  | RS, HS or RS:HS 1:4 | Yes - between the 3 serum-based media |
|  | Hunter E.S., III, Balkan W., and Sadler T.W. (1988). Improved growth and development of presomite mouse embryos in whole embryo culture**.** *J Exp. Zool.*  **245:** 264-269. | E7.5-9.5 | No |  |  | MS: RS: Tyrode’s 3:0:1, 2:1:1, 1:2:1, 0:3:1 (1-72 h) +/- then RS: Tyrode’s 3:1 (25-72 h) | Yes - between the 4 serum-based media |
|  | Balkan W., Rooman R.P., Hurst-Evans A., Phillips L.S., Goldstein S., Du Caju M.V.L., and Sadler T.W. (1988). Somatomedin inhibitors from human serum produce abnormalities in mouse embryos in culture**.** *Teratology.* **38:** 79-86. | E8.5-E9.5; E9.5-E10.5 | No |  | RS 3:1 with Tyrode's |  | No |
|  | Sadler T.W., Phillips L.S., Balkan W., and Goldstein S. (1986). Somatomedin inhibitors from diabetic rat serum alter growth and development of mouse embryos in culture**.** *Diabetes* **35:** 861-865. | E8.5-E9.5; E9.5-E10.5 | No |  | RS 3:1 with Tyrode's |  | No |
|  | Priscott P.K. and Ford J.R. (1985). An in vitro model of acetaldehyde metabolism by rodent conceptuses. *In Vitro Cell Dev. Biol*. **21:** 88-92. | E10-E11 | No |  | RS 1:3 with DMEM + pen/strep |  | No |
|  | Fisher D.L. and Martinez de Villareal, L. (1982). Effects of hormones on postimplantation mouse embryos in vitro. II. Progesterone and estrogen**.** *J Exp. Zool.* **224:** 205-210. | E9.5-E11; E11.5-E13 | No |  |  | FBS 1:1 with Waymouth's + insulin | No |
|  | Sadler T.W. and New D.A.T. (1981). Culture of mouse embryos during neurulation**.** *J. Embryol. Exp. Morphol.* **66:** 109-116. | E8.5-E10.5 | No |  | RS 100% or RS 1:1 with DMEM or Waymouth's |  | Yes - against in vivo developed embryos |
|  | Tam P.P. and Snow M.H. (1980). The in vitro culture of primitive-streak-stage mouse embryos. *J Embryol. Exp. Morphol.* **59:** 131-143. | E6.5-E8.5; E7.5-E8.5 | No |  | RS 100% or RS 1:1 with DMEM | FBS, NCS, MS, RS; 100% or 1:1 with DMEM | Yes - against in vivo developed embryos, against other sera, and between RS with/without 50% dilution by DMEM |
|  | Galloway S.M., Perry P.E., Meneses J., Nebert D.W., and Pedersen R.A. (1980). Cultured mouse embryos metabolize benzo[a]pyrene during early gestation: genetic differences detectable by sister chromatid exchange**.** *Proc. Natl. Acad. Sci U. S. A* **77:** 3524-3528. | E7.5-E9; E8.5-E10 | No |  |  | FBS: NCS: Eagle's 1:1:18 | No |
|  | Sadler T.W. (1979). Culture of early somite mouse embryos during organogenesis**.** *J. Embryol. Exp. Morphol.* **49:** 17-25. | E8.5-E10.5 | No |  |  | RS, FBS or FBS 1:1 with Weymouth's | Yes - against in vivo developed and FBS- cultured embryos |
|  | Agnish N.D. and Kochhar D.M. (1976). Direct exposure of postimplantation mouse embryos to 5-bromodeoxyuridine in vitro and its effect on subsequent chondrogenesis in the limbs**.** *J Embryol. Exp. Morphol.* **36:** 623-638. | E10.5-E11.5 | No |  |  | FBS 1:1 with Waymouth's | No |
|  | Kochhar, D.M., 1975. The use of in vitro procedures in teratology. *Teratology.* **11,** 273-287. | E11.5-E12.5; E12.5-E13.5 | No |  |  | FBS 1:1 with Weymouth's | No |
|  | Clarkson S.G., Doering J.V., and Runner M.N. (1969). Growth of postimplantation mouse embryos cultured in a serum-supplemented, chemically defined medium**.** *Teratology.* **2:** 181-185. | E8.5-E9.5 | No |  | RS (commercial) 1:4 or 1:9 with Waymouth's |  | Yes - between dilutions of RS and against in vivo developed embryos |

* Abbreviations: CMRL, Connaught Medical Research Laboratories; DMEM, Dulbecco’s Modified Eagle’s Medium; FBS, fetal bovine serum; HBSS, Hank’s Balanced Salt Solution; HS, human serum; KSR, Knock-out Serum Replacement; MS, mouse serum; NCS, newborn calf serum; pen/strep, penicillin and streptomycin; RS, rat serum.

** Unless otherwise stated, rat serum was produced specifically for embryo culture in the research laboratory. ‘Commercial’ rat serum was purchased from a supplier.
