# Supplementary material for: Mouse whole embryo culture: Evaluating the requirement for rat serum as culture medium
Source: Birth Defects Res. 2019 Jun 24;111(16):1165–77. doi: 10.1002/bdr2.1538 (PMC6778057; doi:10.1002/bdr2.1538)
Supplement: Supplementary file 2 — Table S2 Comparison of the amino‐acid compositions of DMEM and GMEM + defined supplements (DS), as used to dilute rat serum for whole embryo culture [file BDR2-111-1165-s002.docx]

**Table S2. Comparison of the amino-acid compositions of DMEM and GMEM + Defined Supplements (DS), as used to dilute rat serum for whole embryo culture**

|  |  | **GMEM + DS (mg/L)** | **DMEM (mg/L)** |
| --- | --- | --- | --- |
| **Essential** | L-Histidine HCl-H_2_O | 21.0 | 42.0 |
| **amino-acids** | L-Isoleucine | 52.4 | 105.0 |
|  | L-Leucine | 52.4 | 105.0 |
|  | L-Lysine HCl | 73.1 | 146.0 |
|  | L-Methionine | 15.0 | 30.0 |
|  | L-Phenylalanine | 33.0 | 66.0 |
|  | L-Threonine | 47.6 | 95.0 |
|  | L-Tryptophan | 8.0 | 16.0 |
|  | L-Valine | 46.8 | 94.0 |
| **Non-essential** | L-Alanine | 8.5 | - |
| **amino-acids** | L-Arginine HCl | 42.0 | 84.0 |
|  | L-Asparagine | 13.2 | - |
|  | L-Aspartic acid | 13.3 | - |
|  | L-Cystine 2HCl | 31.3 | 63.0 |
|  | L-Glutamine | 712.0 | 580.0 |
|  | Glycine | 15.0 | 30.0 |
|  | L-Proline | 11.5 | - |
|  | L-Serine | 21.0 | 42.0 |
|  | L-Tyrosine | 36.0 | 72.0 |
